# Case Report: A rare case of choledochal cyst

**DOI:** 10.12688/f1000research.123930.1

**Published:** 2022-08-10

**Authors:** Wasiq Bin Tariq, Anu Radha Twayana, Neela Sunuwar, Azwar Anjum, Sulav Deo, Sushil Rayamajhi, Amit Singh

**Affiliations:** 1 Emergency Medicine, Kulhudhuffushi Regional Hospital, Kulhudhuffushi, 02110, Maldives; 2 Internal Medicine, Bhaktapur Hospital, Bhaktapur, 44800, Nepal; 3 Internal Medicine, Suraksha Hospital, Biratnagar, 56613, Nepal; 4 Internal Medicine, Swacon International Hospital, Kathmandu, 44600, Nepal; 5 Health Emergency Intervention, Consultant Surgeon, World Health Organization, Nepal, Kathmandu, 44600, Nepal

**Keywords:** biliary cysts, biliary drainage, cholangiography, choledochal cyst type 4, hepaticojejunostomy

## Abstract

**Background:** Choledochal cysts are dilated portions of the biliary tract that account for 1% of all benign biliary diseases. It is prevalent among Asian and female populations and the incidence is 1:100,000–150,000. Among the different types, only 15–35% of all choledochal cysts are type IV cysts, with type I being the most common representing 50–80%. Clinical presentation and therapy of biliary cysts (BC) differ depending on the type.

**Case:** We present a case of a 2-year-old male who presented with non-specific symptoms of multiple episodes of vomiting. Laboratory investigations revealed raised alkaline phosphatase and gamma-glutamyl transpeptidase. His symptoms of acute pancreatitis were resolved with conventional therapy. Ultrasonography of the abdomen showed intra and extra-hepatic cystic biliary tree dilatation suggestive of choledochal cyst Type IV A.

**Conclusions:** Choledochal cysts present with clinical features varying with age and anatomical variants and can pose challenges in management that can be addressed by surgery to avoid further complications.

## Introduction

Choledochal cysts are intrahepatic, extra hepatic or both biliary tree dilatations. The incidence in the Western population is 1 in 100,000–150,000 live births; in Asian populations, 1 in 1,000–13000, ^1^^,^^2^ Japan has two-thirds of all cases. There is predominance in the female population, which is more common in the first decade. Type I is the most common. ^3^ Overall, 80% of cases are infants and young children. ^4^ Clinicopathological characteristics vary depending on their age. Abdominal discomfort, jaundice, and an abdominal mass are the most common symptoms in children. Adults, on the other hand, frequently present with biliary or infectious problems and are more likely to progress to choledocholithiasis and malignant transformation. ^2^ Infants tend to present with painless jaundice, hepatomegaly, and acholic stools, a condition mistaken for biliary atresia, hepatic fibrosis, or cirrhosis. Ultrasonography, endoscopic retrograde cholangiopancreatography (ERCP) and magnetic resonance cholangiopancreatography (MRCP) are diagnostic techniques. ^4^ Complete cyst excision and laparoscopic Roux-en-Y hepaticojejunostomy is safe for both infants and children. ^2^

## Case report

**Figure 1.**
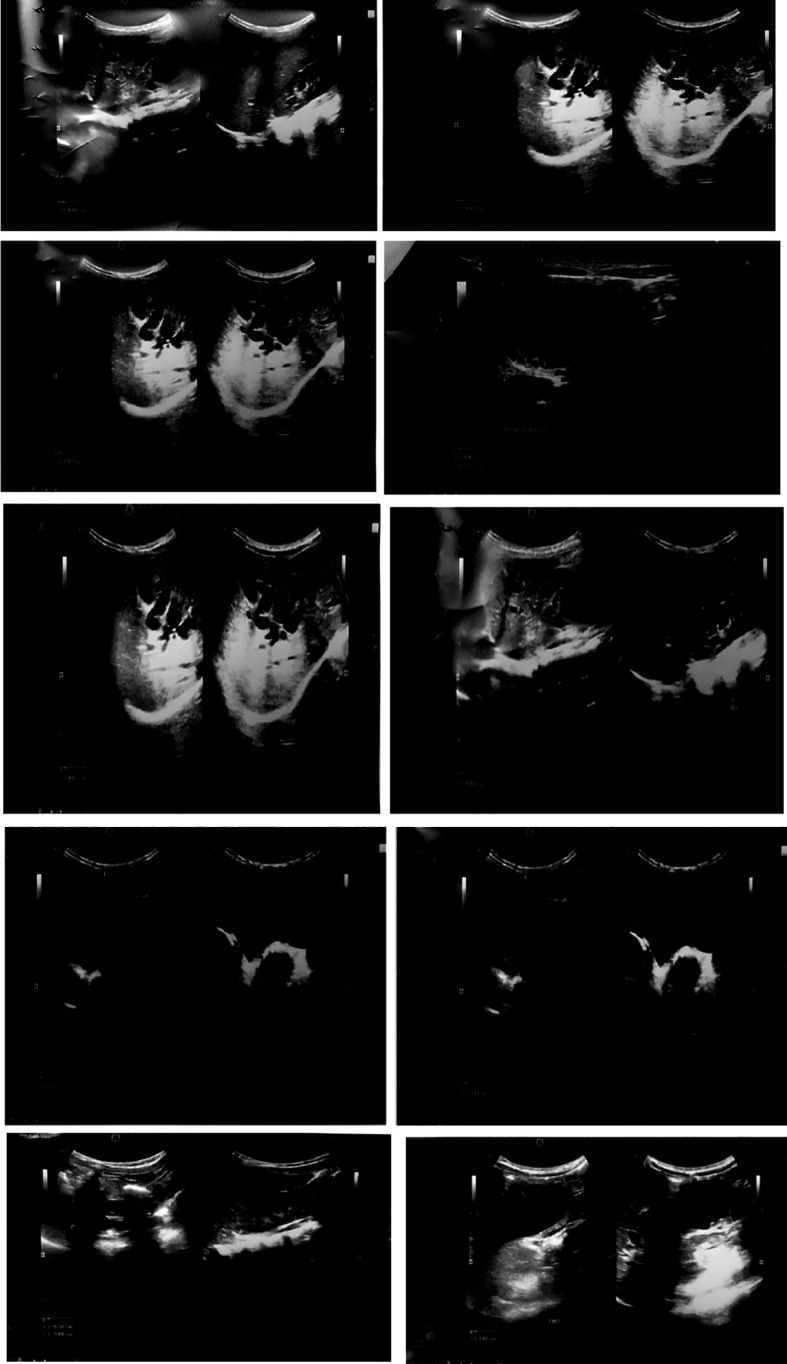
Multiple dilatation of both intrahepatic and extra hepatic biliary duct in keeping with the Type IV A cysts at ultrasonography.

A 2-year-old Asian male child presented with generalized abdominal pain, fever and excessive vomiting for one week. He was not icteric at any point in time. Abdominal examination revealed epigastric tenderness and guarding. There was no other notable medical history. No reports of biliary cysts in family members could be identified. Further laboratory investigations were done, which revealed a cholestatic pattern in serum liver test (raised alkaline phosphatase and gamma glutamyl transpeptidase). His amylase and lipase levels were increased, and the child was diagnosed and treated for acute pancreatitis. His symptoms improved with conventional treatment. The child was kept nil per oral and given intravenous fluids. Later on, ultrasonography of the abdomen was done, which showed both intra and extra hepatic cystic biliary tree dilatation suggestive of choledochal cyst Type IV A, an incidental finding along with soft stones or sludge in the common bile duct ( Figure 1). Despite the fact that computerized tomography (CT) or magnetic resonance imaging (MRI) would have been the next step in the workup, the parents denied further investigation due to financial reasons. In addition, a genetic study of the patient and family could not be conducted. The child’s parents were advised about the necessity for surgery, which included a partial hepatectomy for removal of the intrahepatic section of the cyst and repair with a broad hilar Roux-en-Y hepaticojejunostomy. However, due to financial constraints, they refused surgery.

## Discussion

**Figure 2.**
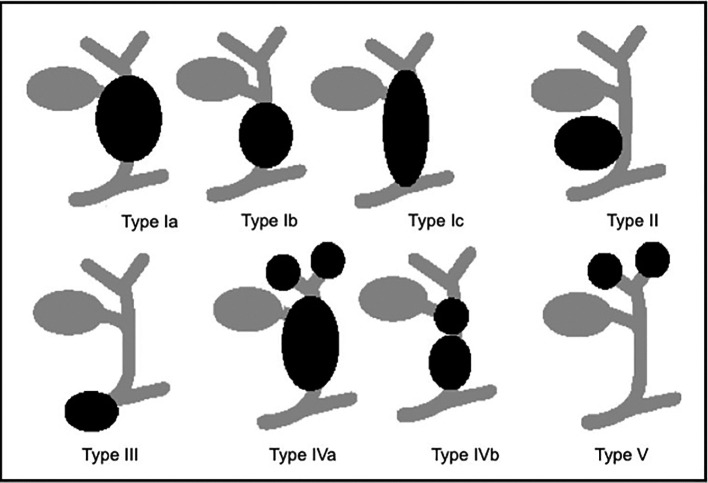
Schematic overview of choledochal cyst (Todani’s classification). This figure has been reproduced from Giha et al., ^9^ under a CC BY-NC-ND 4.0 license.

Choledochal cysts are congenital cystic expansions of the ductus choledochal, the “common channel” that opens into the duodenal lumen. There are five types according to the Todani’s classification ( Figure 2). ^4^

The symptoms are non-specific, such as recurring upper abdominal pain, malaise with nausea and vomiting, which can also be found in biliary tract colic, bile duct abnormalities, or other gastro-intestinal ailments, delaying proper diagnosis. ^5^ Most recent research indicate that nausea, vomiting, and abdominal pain are the most prevalent presenting symptoms in older children (1–18 years of age at presentation), while abdominal mass is predominate in newborns (1 year of age at presentation). ^6^ Similar to the previous studies, the toddler we are reporting on came with a similar finding of multiple episodes of nausea and vomiting and abdominal pain refractory to symptomatic treatment. Furthermore, Badebarin *et al.*, ^6^ Hung *et al.*, ^3^ and Fumino *et al.* ^7^ discovered that abdominal pain was more prevalent in older children than in infants. The study also found that the pattern of choledochal cyst presentation changes from infancy to older children. Infants were more likely than older children to develop nausea, vomiting, and stomach discomfort.

The diagnosis of choledochal cyst is readily established by ultrasound or computed tomography abdomen. ^8^ Similarly, due to recurrent episodes of vomiting, an ultrasound abdomen was advised for the case we are presenting, which revealed both intra and extra hepatic cystic biliary tree dilatation suggestive of choledochal cyst Type IV A along with soft stones or sludge in common bile duct, an incidental finding.

The standard treatments for a symptomatic choledochocele are laparotomy, duodenotomy, and excision of the choledochocele in the sense of internal marsupialization, potentially accompanied with sphincteroplasty. Since the mid-1980s, successful endoscopic therapy, such as endoscopic sphincterotomy or endoscopic cyst excision with a diathermy loop, has been described with increasing regularity. ^5^

There are some limitations to this case study. To begin with, this is a single case, and the finding is unrelated to genetic factors, *i.e.*, it is a spontaneous occurrence. Second, we could not include operation and postoperative details. The child’s parents were advised about the necessity for surgery, however, due to financial constraints, they refused surgery. As a result, the possible predictive factors for postoperative outcomes and complications are unknown. The examination of varied clinical manifestations in the setting of newborns and older children is, nevertheless, a remarkable strength of the current study. Our observations of choledochal cyst symptoms and laboratory abnormalities in this toddler could aid in early detection of the condition and the prevention of late consequences.

## Conclusions

In conclusion, choledochal cysts are a rare finding. Between childhood and adolescence, the pattern of choledochal cyst presentation changes. An abdominal mass is more common in infants, whereas nausea, vomiting, and stomach pain are more common in older children. Surgeons should be aware of the likelihood of anatomical variants in choledochal cysts that fall outside of the standard classification and be prepared for the challenge of management, which may be addressed with careful dissection and correct excision, avoiding additional complications.

## Data availability

### Underlying data

All data underlying the results are available as part of the article and no additional source data are required.

## Consent

Written informed consent for publication of their clinical details and clinical images was obtained from the family of the patient.
