# Supplementary material for: Case Report: A rare case of choledochal cyst
Source: F1000Res. 2022 Aug 10;11:919. [Version 1] doi: 10.12688/f1000research.123930.1 (PMC11079578; doi:10.12688/f1000research.123930.1)
Supplement: Peer review history

# Reviewer report: Reviewer response for version 1

**DOI:** 10.5256/f1000research.136090.r260825

**Published:** 2024-05-08

**Authors:** Simone Maurea

**Affiliations:** 1 University of Naples “Federico II”, Naples, Italy

This interesting clinical case report describes a pediatric patient with choledochal cyst for which the imaging diagnosis is fundamental; the authors illustrate that the patient was evaluated with ultrasound (US),CT and MRI, but only US images are provided.

 Major Point:

 The role of MRCP should be underlined in the discussion section illustrating the possibility to use two technical types of MRCP, the static hydrographic T2-weighted sequence with radial or volumetric scanning and the dynamic post-contrast T1-weighted fat-suppressed sequence using liver specific gadolinium chelates (Multihance or Primovist). In this regard, while the static MRCP depicts all anatomic structures with fluid content, the dynamic MRCP is able to detect only fluid structures corresponding to bile drainage such as a choledochal cyst for which the imaging diagnosis is reached with this methodological approach.

 Minor Point:

 The following references should be cited since report the two technical types of MRCP: 

 1. Maurea S, et. al., 2009 (ref 1).

 2. Maurea S, et. al., 2010 (ref 2)

 3. Maurea S, et. al., 2011 (ref 3)

Are enough details provided of any physical examination and diagnostic tests, treatment given and outcomes?

Yes

Is the case presented with sufficient detail to be useful for other practitioners?

Yes

Is sufficient discussion included of the importance of the findings and their relevance to future understanding of disease processes, diagnosis or treatment?

Partly

Is the background of the case’s history and progression described in sufficient detail?

Partly

Reviewer Expertise:

Abdominal diagnostic imaging

I confirm that I have read this submission and believe that I have an appropriate level of expertise to confirm that it is of an acceptable scientific standard.

## References

[1] : Comparative diagnostic evaluation with MR cholangiopancreatography, ultrasonography and CT in patients with pancreatobiliary disease. *Radiol Med* .2009;114(3) : 10.1007/s11547-009-0374-x 390-402 10.1007/s11547-009-0374-x 19266258

[2] : Magnetic resonance cholangiography with mangafodipir trisodium in Caroli's disease with pancreas involvement. *JOP* .2010;11(5) :460-3 20818116

[3] : Magnetic resonance cholangiography to evaluate biliary tree integrity after cholecystectomy: a case report. *J Dig Dis* .2011;12(3) : 10.1111/j.1751-2980.2011.00500.x 223-5 10.1111/j.1751-2980.2011.00500.x 21615878

# Reviewer report: Reviewer response for version 1

**DOI:** 10.5256/f1000research.136090.r147153

**Published:** 2022-08-11

**Authors:** Bishayeeta Shrestha

**Affiliations:** 1 Department of Oral Pathology, Gandaki Medical College, Pokhara, Nepal

The background seems incomplete. The author could have added the uniqueness of the cases (why your case is unique), the treatment and outcome (why this wasn't possible due to patient's factor should have been included in background).

 Aquedate description was included regarding the diagnostic approaches and treatment plan. But the case report had its own limitation as treatment wasn't possible.

 Sufficient discussion was included of the importance of the findings and their relevance to future understanding of disease processes, diagnosis or treatment. Case presented with sufficient detail to be useful for other practitioners

Are enough details provided of any physical examination and diagnostic tests, treatment given and outcomes?

Partly

Is the case presented with sufficient detail to be useful for other practitioners?

Yes

Is sufficient discussion included of the importance of the findings and their relevance to future understanding of disease processes, diagnosis or treatment?

Yes

Is the background of the case’s history and progression described in sufficient detail?

Partly

Reviewer Expertise:

Maxillofacial Pathology, Immunohistochemistry, medical education

I confirm that I have read this submission and believe that I have an appropriate level of expertise to confirm that it is of an acceptable scientific standard.
